# Trends in the prescription of drugs used for insomnia: an open-cohort study in Australian general practice, 2011–2018

**DOI:** 10.3399/BJGP.2021.0054

**Published:** 2021-08-10

**Authors:** Mumtaz Begum, David Gonzalez-Chica, Carla Bernardo, Amelia Woods, Nigel Stocks

**Affiliations:** Adelaide Medical School, University of Adelaide, Adelaide.; Adelaide Medical School, University of Adelaide, Adelaide.; Adelaide Medical School, University of Adelaide, Adelaide.; Adelaide Medical School, University of Adelaide, Adelaide.; Adelaide Medical School, University of Adelaide, Adelaide.

**Keywords:** benzodiazepines, electronic health records, general practice, insomnia, sleep drug therapy, tranquilising agents

## Abstract

**Background:**

Despite an increase in the prevalence of sleep problems, few studies have investigated changes in the prescribing of drugs that are often used to manage insomnia.

**Aim:**

To explore changes in the pattern of benzodiazepine (BZD), Z-drug (zolpidem, zopiclone), and non-BZD prescriptions.

**Design and setting:**

Open-cohort study comprising 1 773 525 patients (55 903 294 consultations) who attended one of 404 Australian general practices at least three times in two consecutive years between 2011 and 2018.

**Method:**

Data were extracted from MedicineInsight, a database of 662 general practices in Australia. Prescription rates per 1000 consultations, the proportion of repeat prescriptions above recommendations, and the proportion of prescriptions for patients with a recent (within 2 years) recorded diagnosis of insomnia were analysed using adjusted regression models.

**Results:**

Rates of BZD, Z-drug, and non-BZD prescriptions were 56.6, 4.4, and 15.5 per 1000 consultations in 2011 and 41.8, 3.5, and 21.5 per 1000 consultations in 2018, respectively. Over the whole study period, temazepam represented 25.3% of the prescriptions and diazepam 21.9%. All BZD and zolpidem prescriptions declined over the whole study period (annual change varying from −1.4% to −10.8%), but non-BZD and zopiclone prescriptions increased in the same period (annual change 5.0% to 22.6%). Repeat prescriptions that exceeded recommended levels remained at <10% for all medications, except melatonin (64.5%), zolpidem (63.3%), zopiclone (31.4%), and alprazolam (13.3%). In 2018, >50% of Z-drug and melatonin prescriptions were for patients with insomnia. There was an annual increase of 0.8–5.9% in the proportion of prescriptions associated with a recently recorded diagnosis of insomnia.

**Conclusion:**

Overall, BZD prescriptions in Australia declined between 2011 and 2018. However, the prescription of some of these drugs increased for patients with a recently recorded diagnosis of insomnia. This is concerning because of the potential adverse effects of these medications and the risk of dependence.

## INTRODUCTION

Sleep disorders, particularly insomnia, are among the most common, yet frequently overlooked, health problems in the community.^1^^–^2 Chronic insomnia has been associated with a higher risk of obesity, hypertension, heart disease, depression, accidents, impaired productivity, and reduced quality of life.^3^^–^8 The prevalence of sleep problems in Australia increased from 20–35% in 2010 to 33–45% in 2016;^1^^–^2 moreover, in 2016–2017, the total cost of insomnia and other sleep disorders in Australia was estimated to be A$66.3 billion.^3^

Benzodiazepines (BZDs), Z-drugs (that is, a group of sedative drugs that differ from BZD and include zolpidem, zopiclone, and zaleplon), and some antidepressants can be used to treat insomnia and other sleep disorders; however, they are recommended for short-term management and only if non-pharmacological therapies are ineffective.^9^ Long-term use of these medications has been linked to an increased risk of misuse, dependency, drug tolerance, traffic accidents, falls/fractures, impaired quality of life, reduced productivity, hospitalisations, and deaths.^10^^–^15 Of concern, the death rate in Australia that is potentially associated with BZD use increased from 1.9 per 100 000 people in 2008 to 3.5 per 100 000 people in 2018;^16^ a similar pattern of increased deaths associated with BZD use was reported in the US between 1996 and 2013.^13^ This reflects that the issue does not affect Australia alone.

There is a discrepancy in global BZD prescribing and dispensing prevalence due to variations in data sources and years investigated. For example, studies using prescribing data demonstrated that BZD prescribing prevalence among adults ranged from 2.2% of all prescriptions written by GP registrars in Australia (2011–2013)^17^ to 15% of patients attending general practices in Massachusetts (2011–2012).^18^ Studies using filling/dispensing data reported that the proportion of the adult population that received BZDs ranged between 8.1% (Canada, 2016)^19^ and 14.2% (Spain, 2015).^20^

The prescribing and dispensing of BZDs and other related drugs has increased in high-income countries, such as the US (1996–2013)^13^ and Sweden (2006–2013),^21^ but has declined in Canada (2001–2016, drug dispensation data),^19^ Spain (2002–2015, prescription billing data),^20^ South Korea (2009–2013, health insurance data),^22^ Ireland (2002–2011, pharmacy claim database),^23^ and England (2000-2015, Clinical Practice Research Datalink).^24^ Moreover, an upsurge in the prescription of Z-drugs and other non-BZDs has been reported in the last decade in Canada,^19^^,^^25^ Ireland,^26^ and other European countries.^24^^,^^27^ In Norway, Z-drugs have become the most prescribed medications for insomnia management.^28^ Although BZD use has been linked with adverse health outcomes, Z-drugs were thought to have a good safety profile due to a shorter half-life and lower residual drowsiness;^29^ however, long-term use of Z-drugs is also associated with negative consequences such as risk of dependence, falls, and fractures.^10^^–^11^,^^30^^–^31

**Table:** How this fits in

Previous evidence about prescribing for insomnia in Australia has come from either small surveys or studies using drug-dispensing data, which lack indication information. To explore the prescribing pattern of drugs commonly used to manage insomnia, this study used a large (nearly 56 million consultations) longitudinal dataset, spanning the period 2011– 2018, extracted from electronic health records of approximately 2700 GPs and 404 general practices across Australia. Overall, benzodiazepine (BZD) prescriptions in Australia declined over the study period; however, non-BZD prescribing, along with repeat prescriptions for Z-drugs and for patients with a recent recorded diagnosis of insomnia, increased. These increases are concerning, given the risk of long-term use, dependence, and adverse health outcomes.

Evidence about drug prescribing for insomnia usually comes from small surveys or studies using data on drug dispensing.^17^^,^^32^^–^33 In the US, data from the National Health and Nutrition Examination Survey (1999–2010) revealed that 3% of adults used prescribed medications for insomnia management.^34^ In Australia, a cross-sectional analysis of data from 645 Australian GP trainees (2010–2013) showed that BZDs constituted 2.2% of all prescriptions, and 28.2% of BZD prescriptions were associated with a diagnosis of insomnia.^17^

Using data from MedicineInsight, a national general practice database in Australia, the study presented here aimed to explore current trends in the prescription of BZDs, Z-drugs, and other non-BZD medications usually prescribed for insomnia management. In addition, it investigated whether repeat prescriptions for these drugs exceeded Australian recommendations, as well as the proportion of these prescriptions that were for patients with a recently (within 2 years) recorded diagnosis of insomnia.

## METHOD

### Setting, study design, and data source

This open-cohort study used longitudinal de-identified data from MedicineInsight, a large-scale primary care Australian database containing electronic health records (EHRs) from approximately 2700 GPs and 662 general practices across Australia (8.2% of all Australian practices).^35^ Australia has a universal healthcare system called Medicare, which is funded by the Australian Government through taxation revenue; it covers GP visits (Medical Benefits Scheme), hospitalisations, and most medication costs (Pharmaceutical Benefits Scheme) for Australian nationals, permanent residents, and people from countries with reciprocal healthcare agreements. Most general practices are privately owned and operated, but the cost of GP consultations in all practices are fully or partially covered by Medicare rebates for clinical services.^36^ The characteristics of practices recruited by MedicineInsight are reflective of all Australian practices.^35^

MedicineInsight extracts de-identified EHRs from all patients attending these participating practices every month, including the exact date when associated diagnoses, reasons for encounter, reasons for prescription, prescribed medications, laboratory results, or clinical assessments were performed or recorded, as well as current data on sociodemographic characteristics. During data extraction, every patient registered with a participating practice receives a unique identification number that allows them to be tracked over time.

### Study population

This study includes all patients of any age or sex who attended the practices on at least three visits in two consecutive years,^35^ as recorded on MedicineInsight between 1 January 2011 and 31 December 2018. Data were only kept for analysis if they were from practices with a consistent number of consultations over time — that is, a ratio <5 between the maximum and minimum number of consultations in the same practice between 2011 and 2018, with no gaps of more than 6 weeks in the previous 2 years in practice data. This strategy was used to reduce the risk of selection bias, as changes in patterns of prescribed medications could reflect abrupt changes in the number of patients in certain practices, rather than changes in prescribing.

Administrative contacts (phone calls, reminders) and duplicated records were excluded — that is, only one consultation per day per patient was retained for analysis. The final sample consisted of 55 903 294 consultations of 1 773 525 patients regularly attending 404 general practices.

### Data extraction

In routine clinical practice, Australian GPs can record clinical data — that is, diagnosis, reason for encounter, reason for prescription — in a patient’s EHR using either precoded fields or free text that may include standard clinical terminology, spelling variations, and misspellings. Prescriptions are recorded using standardised terms. Data on patients’ demographics, clinical information, and prescribed medications were extracted from MedicineInsight using Stata MP (version 15.1).

Prescriptions and the number of repeats of 11 different BZDs approved for use in Australia (temazepam, diazepam, oxazepam, nitrazepam, alprazolam, lorazepam, clonazepam, flunitrazepam, clobazam, midazolam, and bromazepam), two Z-drugs (zolpidem and zopiclone), and four related non-BZDs (amitriptyline, mirtazapine, quetiapine, and melatonin) traditionally used for insomnia management^9^ were extracted from the ‘script item’ dataset, which used either the active ingredient or commercial brand name.^37^

Records of insomnia or sleep issues were extracted from the ‘diagnosis’, ‘reason for encounter’, and ‘reason for prescriptions’ datasets. Algorithms for data extraction included pre-coded terms, synonyms, and misspellings related to these diagnoses, but excluded obstructive sleep apnoea.

### Outcomes

BZD, Z-drug, and related non-BZD prescription rates (per 1000 consultations) were estimated for each year from 2011 until 2018 inclusive. The repeated annual period prevalence was computed by taking the number of prescriptions (those issued, not considering repeats) in a year as the numerator and the total number of consultations in the corresponding year as the denominator. The researchers then calculated the annual proportion of prescriptions that exceeded the number of repeats (that is, the number of prescriptions issued to the same patient for the same drug in any consultation) recommended by the Pharmaceutical Benefits Scheme (PBS) guidelines, namely:

- more than one prescription for BZDs and Z-drugs;
- more than one prescription for melatonin;
- more than three prescriptions for amitriptyline; and
- more than six prescriptions for mirtazapine or quetiapine.^9^^,^^37^

More than one prescription was used for Z-drugs and melatonin, even though the PBS provides recommendations for zopiclone only (government subsidised). There are PBS guidelines for the drugs that are subsidised by the PBS scheme. Zopiclone is subsidised by the government; zoplidem and melatonin are not subsidised by the government. The authors followed the PBS recommendation regarding repeat prescription of zopiclone, and for zolpidem and melatonin as well.

Finally, estimates were calculated for the annual proportion of BZD, Z-drug, and non-BZD prescriptions for patients with a recently recorded insomnia diagnosis — that is, prescriptions for patients for whom a diagnosis of insomnia was identified in the same year or the year preceding that prescription.

### Statistical analysis

Age- and sex-adjusted annual prescribing prevalence of BZDs, Z-drugs, and non-BZDs were estimated using logistic regression. Marginal adjusted rates per 1000 consultations were then computed and presented graphically. A similar approach was used to estimate changes in the proportion of repeat prescriptions that exceeded recommended limits. Logistic regression was also used to analyse the proportions of these medications that were prescribed to patients with a recently recorded diagnosis of insomnia (age and sex adjusted). The annual change in prescription rates, repeat prescriptions, and the prescriptions associated with insomnia diagnosis were estimated using Poisson regression.

Sensitivity analyses were performed to investigate whether any increase in the proportion of prescriptions linked to a recently recorded diagnosis of insomnia was related to surveillance bias (that is, better recording of insomnia in the medical records in more recent years). For that purpose, analyses were repeated considering incident/first-time recorded prescriptions of BZDs and related drugs, incident/first-time recorded insomnia diagnosis, and only including EHRs from patients with recorded data for the whole period (that is, at least one consultation in each year between 2011 and 2018).

All analyses were conducted using Stata/ MP (version 15.1), considering the practice as a cluster and using robust standard errors.

## RESULTS

The sample included 1.7 million patients contributing to a total of 55 903 294 consultations between 2011 and 2018. Females attended 60.0% of all consultations; 40.6% of the consultations occurred among individuals aged ≥65 years, 50.3% were among patients aged 18–64 years, and 9.1% were for those aged <18 years. Missing data on any covariate represented <0.5% of the sample. Of all the consultations over the study period, 3 740 458 (6.7%) were linked to a BZD, Z-drug, or non-BZD prescription; 896 138 (24.0%) of the prescriptions were given to patients with a recent record of insomnia diagnosis.

The most commonly prescribed drugs from those investigated over the whole period were temazepam (25.3%) and diazepam (21.9%), followed by amitriptyline (12.9%), oxazepam (10.9%), mirtazapine (6.8%), quetiapine (4.6%), nitrazepam (3.6%), melatonin (3.3%), and zolpidem (3.3%; data not shown).

**Figure 1.**
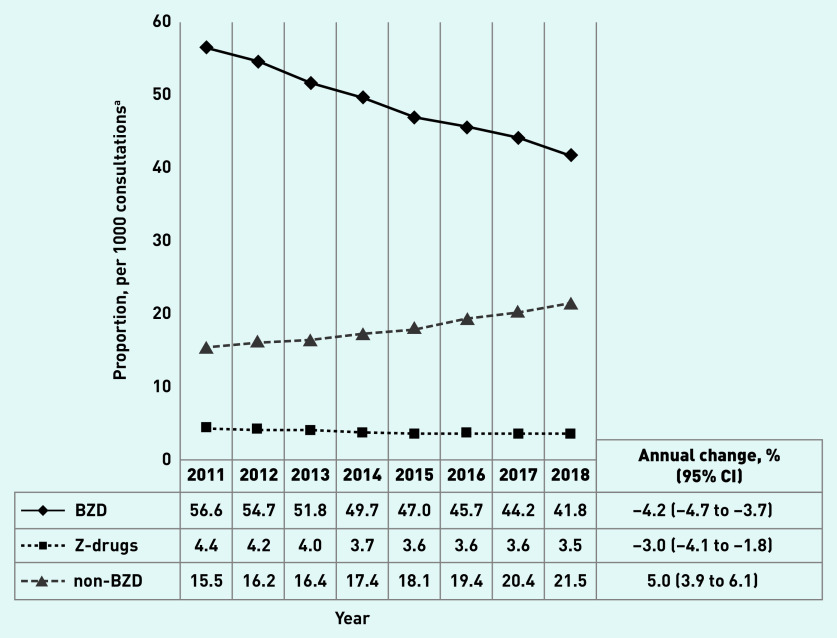
*Overall benzodiazepines, Z-drugs, and non-benzodiazepines prescription rate per 1000 consultations in Australian general practice, 2011–2018 (N = 55 903 294 consultations). ^a^Results adjusted for age and sex. BZD = benzodiazepine.*

Figure 1 shows that the overall BZD prescribing rate declined from 56.6 per 1000 consultations (95% confidence interval [CI] = 53.8 to 59.3) in 2011 to 41.8 per 1000 consultations (95% CI = 39.9 to 43.7) in 2018 (annual change −4.2% [95% CI = −4.7 to −3.7]). The prescribing of Z-drugs changed from 4.4 per 1000 (95% CI = 4.0 to 4.8) in 2011 to 3.5 per 1000 consultations (95% CI = 3.1 to 3.9) in 2018 (annual change −3.0% [95% CI = −4.1 to −1.8]), and non-BZD prescription rates increased from 15.5 per 1000 consultations (95% CI = 14.0 to 17.0) in 2011 to 21.5 per 1000 consultations (95% CI = 20.8 to 22.3) in 2018 (annual change 5.0% [95% CI = 3.9 to 6.1]). Similar trends were identified when the total number of patients, rather than the total number of consultations, was used as the denominator: 2% annual decline in overall BZD and Z-drugs, and 6.2% annual increase in the prescription of non-BZD between 2011 and 2018 (Supplementary Tables S1a and S1b).

**Figure 2.**
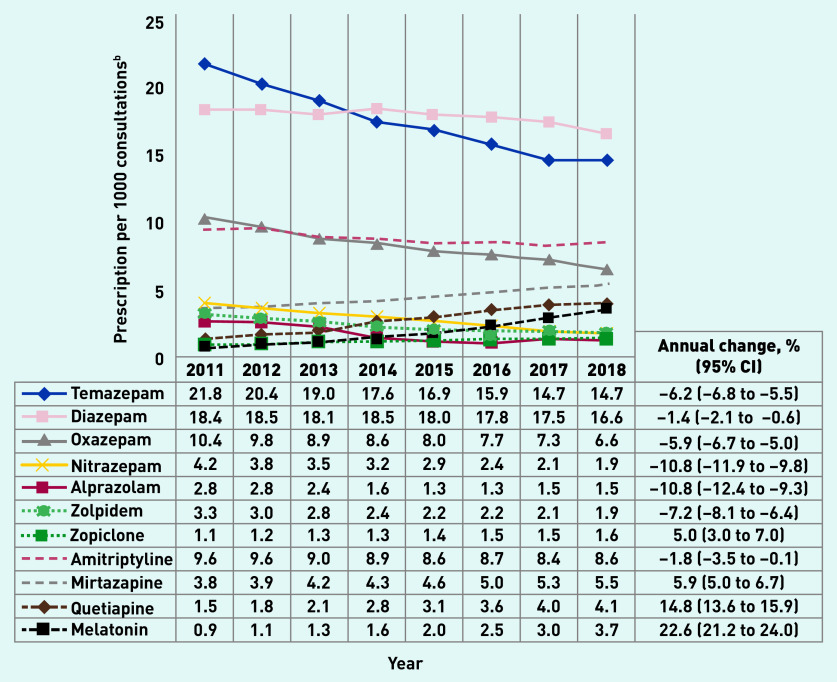
*Benzodiazepines, Z-drugs, and nonbenzodiazepines^a^ prescription rate per 1000 consultations in Australian general practice, 2011–2018 (N = 55 903 294 consultations). ^a^BZD (temazepam, diazepam, oxazepam, nitrazepam, alprazolam); Z-drugs (zolpidem and zopiclone); non-BZD (amitriptyline, mirtazapine, quetiapine, melatonin). ^b^Results adjusted for age and sex. BZD = benzodiazepine.*

Figure 2 shows that the prescribing prevalence (age and sex adjusted) of most BZDs declined between 2011 and 2018, with an annual decrease varying from −1.4% (diazepam) to −10.8% (nitrazepam, alprazolam). Zolpidem and zopiclone showed opposing trends to each other: zolpidem prescriptions had, on average, a 7.2% annual decrease, while zopiclone prescription increased 5.0% per year. With the exception of amitriptyline, all non-BZD prescriptions, and melatonin in particular, increased in the investigated period. Drugs with a prescription rate of <1 per 1000 consultations are presented in Table 1. Crude results showed a similar pattern (Supplementary Table S2).

**Table 1.** Benzodiazepines with prescription rate <1 per 1000 consultations, Australian general practices, 2011–2018 (*N* = 55 903 294 consultations)^a^

**Medication**	**2011**	**2012**	**2013**	**2014**	**2015**	**2016**	**2017**	**2018**	**Annual change, % (95% CI)**
Lorazepam	0.7	0.7	0.7	0.7	0.8	0.8	0.9	1.0	6.4 (4.5 to 8.3)
Clonazepam	0.8	0.8	0.8	0.8	0.8	0.8	0.8	0.8	0.7 (−0.5 to 1.9)
Flunitrazepam	0.3	0.3	0.2	0.2	0.2	0.2	0.1	0.1	−13.2 (−16.0 to −10.4)
Bromazepam	0.2	0.2	0.2	0.2	0.2	0.1	0.1	0.1	−4.9 (−7.6 to −2.2)
Clobazam	0.1	0.1	0.1	0.1	0.1	0.1	0.1	0.1	2.6 (−0.1 to 5.3)
Midazolam	0.0	0.0	0.0	0.0	0.0	0.1	0.1	0.3	51.8 (40.9 to 63.5)

a *Results adjusted for age and sex. CI = confidence interval.*

**Figure 3.**
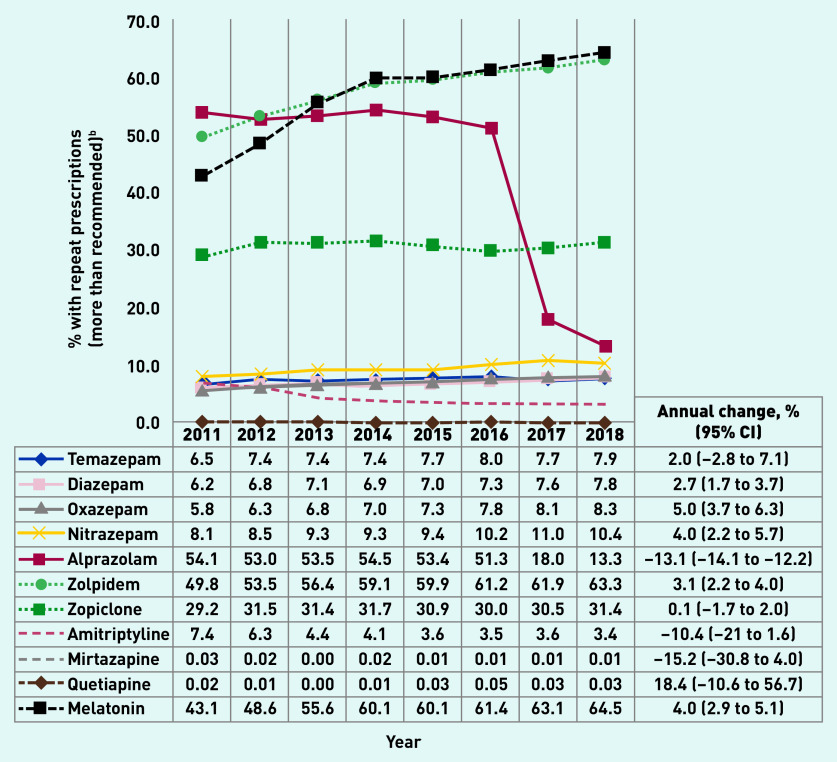
*Proportion of benzodiazepines, Z-drugs, and non-benzodiazepines^a^ scripts written with more repeats than recommended by guidelines, Australian general practices, 2011-2018. ^a^ BZD (temazepam, diazepam, oxazepam, nitrazepam, alprazolam); Z-drugs (zolpidem and zopiclone); non-BZD (amitriptyline, mirtazapine, quetiapine, melatonin). ^b^ Results adjusted for age and sex. Recommended repeats: temazepam, diazepam, oxazepam, nitrazepam, alprazolam, zolpidem, zopiclone, melatonin = zero repeats; amitriplyline = up to two repeats; mirtazapine/quetiapine = up to five repeats. BZD = benzodiazepine.*

Figure 3 depicts age- and sex-adjusted proportions of BZD and related drugs prescribed with repeat prescriptions, above PBS recommendations. Repeat prescriptions above recommended levels for alprazolam showed a substantial reduction between 2016 (51.3%) and 2017 (18.0%). Zolpidem and melatonin showed a similar pattern with more than 60% prescriptions written with repeats in 2018. Zopiclone prescriptions remained steady over time, with ~30% of prescriptions written with repeats. The proportion of prescriptions with repeats above recommended levels remained at <10% for all other medications. Over the whole study period, the highest annual increase (9.9%) was observed for the repeat prescription of non-BZDs, compared with BZDs and Z-drugs (Supplementary Figure S1).

Table 2 shows that Z-drugs and melatonin had the highest proportion of prescriptions for patients with a recently recorded diagnosis of insomnia (>50% in 2018). Prescriptions for temazepam and nitrazepam, in 2018, for patients with a recently recorded insomnia diagnosis was approximately twice as high (38.1% and 40.9% in 2018, respectively) as those for diazepam (19.5%), oxazepam (26.9%), alprazolam (14.7%), or any other non-BZD except melatonin. Except for melatonin, there was an annual increase in the proportion of prescriptions associated with a diagnosis of insomnia, which ranged from 3.2% to 5.9% for all the investigated medications. To address whether the increase in the prescription of these medications for insomnia was due to improved diagnosis recording in recent years, sensitivity analyses were performed that considered ‘incident’ (that is, first time) recorded prescription, incident-recorded insomnia, and including patients with at least one consultation per year between 2011 and 2018. Compared with the main findings, sensitivity analyses showed that the annual increase in the proportion of prescriptions associated with a diagnosis of insomnia was only evident for temazepam, nitrazepam, both Z-drugs, mirtazapine, and quetiapine (Supplementary Table S3).

**Table 2.** Proportion of prescriptions of benzodiazepines, Z-drugs, and non-benzodiazepines with a recorded insomnia diagnosis, Australian general practices, 2011–2018

**Medication**	**Proportion of prescriptions with a recorded insomnia diagnosis, %^a^**								**Annual change, % (95% CI)**
	**2011**	**2012**	**2013**	**2014**	**2015**	**2016**	**2017**	**2018**	
**Benzodiazepines**									
Temazepam	25.3	26.9	29.6	31.4	32.2	33.7	35.4	38.1	5.6 (4.6 to 6.6)
Diazepam	14.0	14.2	15.5	16.5	17.2	17.8	19.0	19.5	5.1 (3.7 to 6.4)
Oxazepam	19.2	20.8	21.5	23.3	23.3	24.0	24.7	26.9	4.2 (2.7 to 5.8)
Nitrazepam	28.0	28.6	31.9	34.5	35.3	37.8	39.8	40.9	5.9 (4.0 to 7.7)
Alprazolam	11.6	11.8	13.6	13.6	14.5	14.8	15.7	14.7	4.0 (1.6 to 6.4)
**Z-drugs**									
Zolpidem	37.9	39.2	43.5	43.4	44.4	46.6	47.6	50.5	3.8 (2.6 to 5.0)
Zopiclone	44.4	46.2	45.0	47.8	49.9	51.5	52.7	54.6	3.2 (1.8 to 4.5)
**Non-benzodiazepines**									
Amitriptyline	11.6	12.8	14.3	14.9	15.0	16.1	16.8	17.2	5.1 (3.3 to 6.7)
Mirtazapine	15.2	15.2	16.9	18.3	18.2	19.0	19.9	20.4	4.3 (3.1 to 5.5)
Quetiapine	14.6	15.5	16.1	16.8	17.3	18.4	19.5	19.9	4.6 (2.9 to 6.3)
Melatonin	48.1	47.8	48.9	49.5	49.5	51.1	50.7	50.7	0.8 (0.0 to 1.7)

a *Results adjusted for age and sex. Insomnia diagnosis recorded in current year or 1 year preceding the prescription. CI = confidence interval.*

## DISCUSSION

### Summary

In this large, open-cohort study, the researchers explored trends in the prescribing of BZDs, Z-drugs, and non-BZDs using a nationwide sample of Australian general practices. Four main findings can be highlighted.

Consistent with international trends,^19^^,^^22^^–^24 BZD prescriptions have reduced over time, especially for nitrazepam and alprazolam; there has been a rise in the prescription of non-BZDs (melatonin in particular) and zopiclone, but not zolpidem. Nonetheless, temazepam and diazepam are still two or three times more likely to be prescribed than any of the other investigated medications, accounting for half of all BZD, Z-drug, and non-BZD prescriptions.

More than half of medications not funded by the PBS (melatonin and zolpidem are not funded) were provided with one or more repeats, as well as a third of prescriptions for the PBS-funded zopiclone. The prescription of multiple repeats for alprazolam reduced dramatically in 2017; for all other drugs, <10% of prescriptions had repeats that exceeded the recommended levels.^9^^,^^37^

More than half of Z-drug or melatonin prescriptions in 2018 were for patients with a recently recorded diagnosis of insomnia (34 551 of 67 443 prescriptions for the three drugs combined). However, in absolute terms for each individual drug, temazepam (53 469 out of 141 994 prescriptions) had the highest number of prescriptions written for patients with a recently recorded insomnia diagnosis, followed by diazepam (30 062 out of 154 562 prescriptions).

Finally, overall BZD prescribing reduced, but the prescription of some BZDs for insomnia increased over the study period.

### Strengths and limitations

There are strengths to this study, namely its use of a large, longitudinal national general practice database of 8 years, and data being recorded by GPs, not self reported.

Some limitations should also be recognised. The indications for prescribing are not commonly recorded and, as such, conditions may not be directly linked with BZD prescribing. In addition, the completeness and accuracy of recorded information may vary between GPs. This study was also based on the number of written prescriptions, but it is not known whether the patient filled the prescription and used the medication; therefore, the estimations may represent an overestimation of actual medication use. Similarly, it has not been possible to account for patients visiting other practices and obtaining and using additional prescriptions for BZDs. However, the researchers believe that these factors would only affect a small proportion of patients because they used data of regular patients with three visits to the same practice in consecutive 2 years. Lastly, data can be recorded as free text and algorithms for data extraction may lead to under/overestimation (measurement bias); however, the findings presented here regarding trends are consistent with previous reports/papers.^32^

### Comparison with existing literature

Prescribing rates were estimated using the total number of consultations as the denominator and are not directly comparable with other studies that used total population, person years, or other denominators. However, sensitivity analyses using patients as the denominator showed that the prevalence of BZD prescribing in Australian general practice in 2018 (89.3 per 1000 population) was lower than that for Ireland (166.1 per 1000 population, 2015),^26^ but higher than that for Canada (72.4 per 1000 population, 2011/2012).^25^ Although the burden and impact of mental health disorders are similar across these countries,^38^ study methodologies, different anti-BZD campaigns across countries, adherence to guidelines, and different healthcare-seeking behaviour may explain these prescribing discrepancies.^27^^,^^39^^–^40

Overall, an annual decline of 4.2% in the prescription of BZDs was identified for the period 2011–2018. Similar declining trends were previously reported in Australia (2000–2011, 1992–2011)^32^^,^^41^ and other countries.^22^^–^23^,^^25^^–^27 In Europe, a decline in BZD prescription ranging from 4% to 26.5% was observed when analysing EHRs (2001–2009)^39^ or drug-dispensing data (2005–2015).^26^

Campaigns to reduce long-term BZD prescriptions and use of Z-drugs in nine European countries showed limited efficacy, except when subsidised alternatives were made available, such as the availability of prolonged-released melatonin.^27^ It was reported that, in Australia, the introduction of the government-funded medication reviews programme in 2001 may have been responsible for an initial decline in BZD prescribing;^42^ however, according to the findings presented here, the release of new Australian guidelines^9^ in 2015 to address the concerns regarding BZD harms and misuse was not followed by any substantial change in trends.

In general, there was a small increase in the proportion of BZDs prescribed, with repeat prescriptions exceeding recommended levels. Nonetheless, the proportion of prescriptions with multiple repeats remained at <10% for most BZDs and non-BZDs; this was expected, given that multiple repeats of these drugs are only allowed for people in certain situations or with specific conditions (for example, in palliative care or for patients with late-stage malignant neoplasia).^37^ The large reduction in multiple repeats for alprazolam could be attributed to its rescheduling in 2014 (addiction category), with further restrictions introduced in 2017 (pack size reduced from 50 to 10, no repeats allowed).^43^ Other Australian studies also reported decline in alprazolam use after the resheduling.^44^^–^45

Z-drugs are primarily indicated for insomnia, and their use has increased in high-income countries, including Australia.^25^^–^27^,^^46^^–^47 However, in the study presented here, 45% of Z-drug prescriptions did not have any indication of recently recorded insomnia diagnosis. Similarly, a high proportion of prescriptions for BZDs or Z-drugs without any recorded indication were reported in a study using seven European EHR databases.^39^ Not all fields that may contain the diagnosis are extracted by MedicineInsight because of confidentiality issues; one example of this is progress notes. It is also possible that these medications were prescribed for other conditions associated with insomnia (for example, depression or anxiety), or insomnia itself may have been recorded before the allowable window used in the study presented here.^39^ Further analysis showed that, in 2018, 76.6% of Z-drug prescriptions from the cohort in this study were for people with any record of insomnia, and 11.1% were for people who had any record of ill mental health between 2011 and 2018.

### Implications for practice

Overall, BZD prescriptions in this study cohort in Australia declined between 2011 and 2018, but the prescription of some BZDs for insomnia management has increased. Despite the observed decline, current BDZ prescribing rates are higher than expected, based on current recommendations. This perspective should be viewed in the context of a 70% increase in the number of deaths involving BZDs between 2009 and 2018 in Australia.^48^ The proportion of prescriptions with multiple repeats for the government-subsidised zopiclone remained steady at ~30%, whereas multiple prescriptions for the non-subsidised zolpidem increased from 50% to 63% between 2011 and 2018. The finding that more than half of non-PBS-funded medications (that is, melatonin and zolpidem) were provided with repeat prescriptions suggests long-term treatment. Although Australian guidelines suggest a similar level of caution when prescribing zopiclone and zolpidem,^9^ explicit recommendations seem necessary regarding repeated prescriptions for zolpidem. Z-drugs are not innocuous and are also associated with increased risk of dependency, drug tolerance, falls/fractures, and poor quality of life.^10^^,^^11^^,^^30^ Although melatonin is considered safer than BZDs or Z-drugs, its long-term effects have not been fully explored.^49^

In spite of declining trends in overall BZD and Z-drug prescriptions, the increasing proportion of them provided to patients with an insomnia diagnosis is concerning, as it could increase the likelihood of dependence. The findings reported here are consistent with the reported rise in the prevalence of sleep issues in Australia during the past decade;^1^^,^^2^ they highlight the need to improve access to recommended treatments for insomnia management (for example, cognitive behavioural therapy) and reduce sedative-hypnotic prescribing.^50^

The findings presented here also suggest that, if restrictions on the number of repeats for some medications on private prescriptions were introduced, this might discourage long-term prescribing.^9^^,^^37^ Although there are clear regulations for the prescription of drugs included in the government-subsidised PBS scheme, there is a paucity of explicit guidance for non-PBS drugs;^37^ therefore, explicit recommendations regarding repeated prescriptions for non-subsidised drugs seem necessary.

Other interventions may also help reduce the number of BZD prescriptions sought by patients and prescribed by GPs, such as reducing pack sizes where possible, increasing the availability of non-pharmaceutical interventions for insomnia management,^51^ and introducing real-time prescription monitoring to identify patients who visit multiple doctors for prescriptions.^52^
